# Immobilized Wild-Type and Mutant L‑Alanine Dehydrogenase from *Thermus thermophilus* Coupled with Formate Dehydrogenase for Asymmetric Synthesis of Noncanonical Amino Acids

**DOI:** 10.1021/acsomega.6c04318

**Published:** 2026-08-19

**Authors:** Ğarip Demir, Deniz Yildirim, Barış Binay

**Affiliations:** † Department of Molecular Biology and Genetics, Gebze Technical University, Gebze, Kocaeli 41400, Türkiye; ‡ Department of Chemical Engineering, Faculty of Engineering, 37506 University of Cukurova, Adana 01130, Türkiye; § Department of Bioengineering, Faculty of Engineering, Gebze Technical University, Gebze, Kocaeli 41400, Türkiye; ∥ BAUZYME Biotechnology Co., Gebze Technical University Technopark, Gebze, Kocaeli 41400, Türkiye

## Abstract

The asymmetric synthesis of noncanonical amino acids (ncAAs) is of increasing interest due to their importance as precursors for pharmaceuticals, enzyme inhibitors, and functional peptide building blocks. In this study, wild-type and Tyr92Ser mutant l-alanine dehydrogenases from *Thermus thermophilus* (*Tt*AlaDH) (WT and Mut, respectively) were immobilized using three different strategies: covalent attachment on Eupergit C 250 L (EC250L), cross-linked enzyme aggregates (CLEAs), and entrapment in poly­(vinyl alcohol) (PVA) gel. Following a comparison of immobilization yield and recovered activity, the resulting preparations were characterized based on their optimum pH/temperature, thermal stability, and kinetic parameters. While covalent immobilization on EC250L and CLEA formation achieved high yields (>90%), they suffered from low recovered activity (≈5–12%). In contrast, PVA entrapment provided full immobilization yield while preserving near-native activity (95–100%). The WT and Mut preparations were individually coimmobilized with *Chaetomium thermophilum* formate dehydrogenase (*Ct*FDH) in PVA gel to establish an integrated NADH regeneration system. The coimmobilized WT and Mut preparations enabled one-pot reductive amination of α-keto acids into enantiopure L-amino acids with excellent stereoselectivity (99% ee). Under optimized enzyme ratios and reaction conditions, l-alanine was obtained in at least 83% yield. Notably, the Mut preparations displayed enhanced selectivity toward bulkier substrates compared to the WT preparation, producing noncanonical l-norvaline and L-norleucine amino acids with yields of up to 47.5% and 74.8%, respectively. The coimmobilized PVA-Mut/*Ct*FDH preparation retained high activity after multiple reuse cycles. This study provides a promising and scalable biocatalytic platform for the sustainable synthesis of l-alanine-derived noncanonical amino acids.

## Graphical abstract

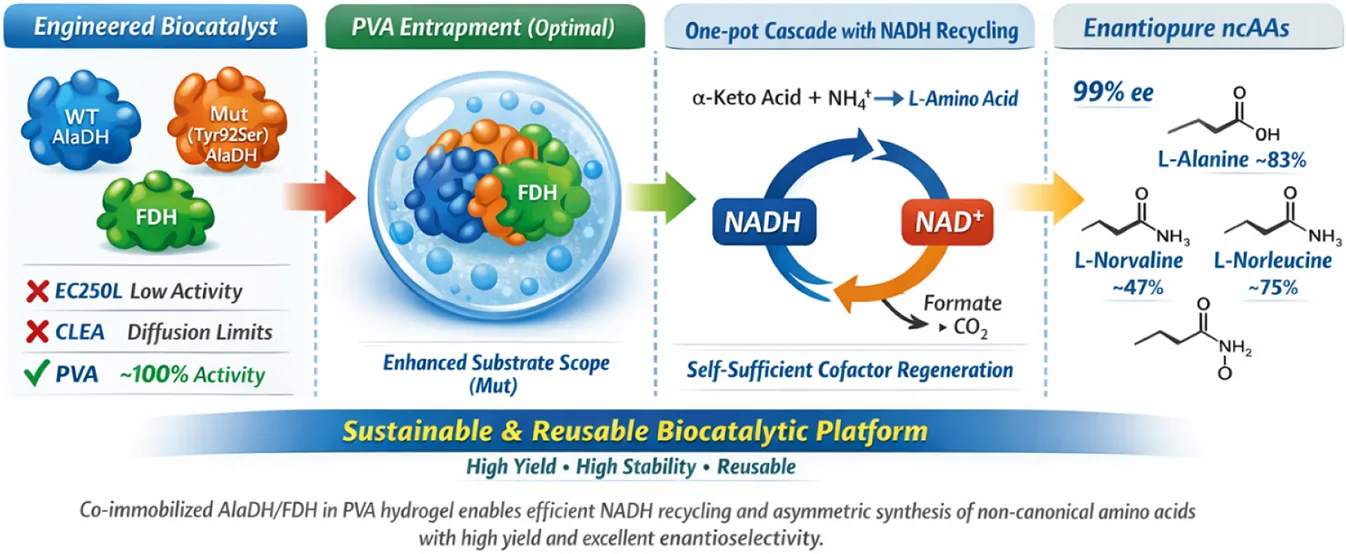

## Introduction

The increasing demand for sustainable, efficient, and highly selective chemical processes has positioned biocatalysis at the forefront of modern industrial biotechnology.^1,2^ As the pharmaceutical, fine-chemical, food additive, and cosmetics industries, as well as academic research seek more efficient routes to use and synthesize complex molecules,^3^ the synthesis of ncAAs has gained significant focus in recent research.^4^ While standard proteinogenic amino acids are limited to the 20 genetically encoded amino acids, there are over 800 naturally occurring ncAAs, predominantly produced by plants and fungi through specialized metabolic pathways, and countless ncAAs designed and synthesized in research settings with diverse applications.^5–13^ Hence, the synthesis of enantiopure chiral ncAAs remains a significant challenge. Conventional chemical methods for ncAAs production are often marred by harsh reaction conditions, the use of toxic substances, and the generation of environmental pollution.^14,15^ In contrast, enzymatic synthesis offers a greener and more efficient alternative, typically avoiding protective groups and proceeding under mild, aqueous conditions with high stereoselectivity.^16,17^ A wide range of enzymes has been applied to the asymmetric synthesis of ncAAs, comprising amino acid dehydrogenases (AADHs), aminotransferases, hydroxymethyltransferases, nitrilases, hydantoinases, and various redox enzymes.^16–18^ However, the transition from traditional chemical synthesis to biological production faces significant hurdles. To bridge this gap, modern research focuses on the synergistic application of protein engineering, enzyme immobilization, and the design of artificial enzyme cascades.^16,18,19^ Among these enzymes, NAD­(H)-dependent l-alanine dehydrogenases (l-AlaDHs) have attracted particular attention due to their ability to catalyze reductive amination reactions. Moreover, l-AlaDHs have a wider range of α-keto acid substrates, catalyzing reductive amination reactions with high enantioselectivity and yield.^20,21^ However, these enzymes require expensive nicotinamide cofactors (e.g., NADH) in stoichiometric quantities, which imposes economic restrictions on industrial viability. Consequently, efficient cofactor regeneration systems are mandatory.^22^

To improve industrial applicability, enzymes are often enhanced through protein engineering and immobilization strategies. Protein engineering improves properties such as thermostability and catalytic activity through amino acid substitutions, while immobilization converts soluble enzymes into reusable biocatalysts with enhanced operational stability and recovery efficiency.^1,23^ Although l-AlaDHs have been applied to ncAA synthesis, studies on their immobilization remain limited.

Ideal immobilization carriers should possess properties such as biocompatibility, chemical stability, and cost-effectiveness.^24,25^ In this study, EC250L, CLEAs, and poly­(vinyl alcohol) (PVA) were selected as immobilization systems. EC250L enables stable covalent enzyme attachment with high mechanical stability;^26–28^ CLEAs provide carrier-free immobilization through enzyme cross-linking;^24^ and PVA serves as a biodegradable and hydrophilic matrix with favorable mechanical properties for enzyme entrapment.^25^

Previous studies of immobilized amino acid dehydrogenases have primarily focused on enzyme stabilization and l-alanine production.^29–32^ In addition, fusion or coupled dehydrogenase systems with formate dehydrogenase have been investigated for cofactor regeneration applications.^30^ However, comprehensive studies integrating protein engineering, immobilization strategy comparison, cofactor regeneration, and asymmetric synthesis of noncanonical amino acids (ncAAs) remain limited. Previously, l-AlaDHs from different microorganisms were cloned, heterologously expressed, and purified by our group.^20,33,34^ Furthermore, wild-type and Tyr92Ser mutant l-alanine dehydrogenases from *Thermus thermophilus* (*Tt*AlaDH) were developed to enhance reductive amination activity toward l-alanine-derived ncAAs.^20^ Although these free enzymes were previously characterized, their industrial applicability has not yet been explored. Therefore, this study investigates the immobilization of WT and Mut *Tt*AlaDH preparations using covalent attachment on EC250L, CLEA formation, and PVA entrapment. The resulting free and immobilized preparations were characterized in terms of the optimum pH and temperature, thermal stability, and kinetic parameters. The most active immobilized preparations were subsequently coimmobilized with NADH-dependent *Chaetomium thermophilum* formate dehydrogenase (*Ct*FDH) to establish an integrated cofactor regeneration system for the synthesis of l-alanine-derived ncAAs, including l-norvaline, L-norleucine, and L-2-aminobutyric acid. By combining protein engineering with multienzyme coimmobilization, this work provides a comprehensive investigation into efficient, reusable, and industrially relevant biocatalytic systems for sustainable ncAA synthesis.

## Materials and Methods

### Materials

Unless otherwise stated, all chemicals were purchased from Sigma-Aldrich (St. Louis, MO, USA). *Escherichia coli* BL21 (DE3) cells used for enzyme expression were obtained from Invitrogen (Waltham, MA, USA). Plasmid vectors encoding *Tt*AlaDH and *Ct*FDH were provided by the Gebze University Enzyme Research Center (GERC, Kocaeli, Türkiye). All culture media were prepared using reagents obtained from LABM (Bury, UK). The Ni-NTA HisTrap column was purchased from Cytiva.

### Methods

#### Expression, Purification, and Activity Assay of WT and Mut

The expression and purification of WT and Mut were achieved by Demir et al.^20^ The reductive amination activity of *Tt*AlaDH was measured according to Dedekayoğulları et al.^34^ The reaction mixture consisted of 850 μL of Tris–HCl buffer (50 mM, pH 8.5) containing 2 M NH_4_Cl, 50 μL of free *Tt*AlaDH (1 mg protein/mL) or 10 mg of immobilized *Tt*AlaDH, 100 μL of 10 mM sodium pyruvate, and 100 μL of 1 mM NADH. The change in NADH concentration was monitored at 340 nm for 5 min. The control group contained 900 μL of Tris–HCl buffer (50 mM, pH 8.5) containing 2 M NH_4_Cl, 100 μL of 10 mM sodium pyruvate, and 100 μL of 1 mM NADH. One unit of *Tt*AlaDH activity was defined as the amount of enzyme that catalyzes the formation of 1 μmol NAD^+^ per minute under the assay conditions.

#### Immobilization of WT and Mut

Immobilization of WT and Mut preparations onto EC250L was performed based on the method proposed by Mateo et al.^35^ Briefly, 9.0 mL of purified enzyme solution (1.0 mg/mL in 50 mM pH 7.0 phosphate buffer) was added to 1.0 g of EC250L support, and the mixture was gently shaken in a water bath at 25 °C for 24 h. The obtained immobilized preparations were washed with 50 mM pH 7.0 phosphate buffer until no protein remained in the filtrate, and then stored at 5 °C. The immobilized WT and Mut preparations were named EC250L-WT and EC250L-Mut, respectively.

The immobilization of WT and Mut preparations as CLEA was performed according to Yildirim et al.^36^ Solid ammonium sulfate was added to 5 mL of pure enzyme solution (1 mg protein/mL) at saturation levels of 30%, 40%, or 50%, followed by 1.5 mL of 25% glutaraldehyde solution. The mixture was incubated at 5 °C for 24 h with gentle shaking. The immobilized CLEA preparations were then washed with pure water until no protein remained in the filtrate. The resulting immobilized enzymes were stored at 5 °C until use. The immobilized WT and Mut preparations were designated as CLEA-WT and CLEA-Mut, respectively.

The immobilization of WT and Mut preparations in PVA gel was carried out using the method suggested by Toprak et al.^37^ Briefly, 10 g of solid PVA was added to 80 mL of water and heated at 90 °C until the polymer was completely dissolved. After cooling the solution to room temperature, a 3-mL aliquot of the PVA solution was mixed with 1 mL of WT and/or preparation (1 mg protein/mL). 0.1-mL samples were taken from the mixture and pipetted onto a plastic surface. Then, the samples were left at 5 °C for 12 h to complete gel formation, and the resulting immobilized WT and Mut preparations were stored at 5 °C until use. The immobilized WT and Mut preparations were named PVA-WT and PVA-Mut, respectively.

All immobilized preparations were washed thoroughly with a phosphate buffer and distilled water until no protein was detected in the washings. The protein concentration in the filtrates was determined using the method of Lowry et al.,^38^ ensuring that immobilization was complete and no leaching occurred. The resulting preparations were stored at 5 °C until further use.

The efficiency of the immobilization processes was evaluated through yield and activity recovery calculations. The immobilization yield (IY%) was calculated using the following formula, where *P_t_* represents the total protein added for immobilization, and *P_s_* represents the protein measured in the filtrate:

1 $$IY\%=\frac{\left(P_{t}-P_{s}\right)}{P_{t}}\times 100$$

The recovered activity (RA%) after immobilization was determined using the formula below, where *A_i_* denotes the activity of the immobilized enzyme and *A_t_* denotes the total initial WT and/or Mut activity:

2 $$RA\%=\frac{A_{i}}{A_{t}}\times 100$$

#### Characterization of Free and Immobilized WT and Mut

To determine the optimum pH, activity measurements for both free and immobilized *Tt*AlaDH preparations were performed at 40 °C across a pH range of 7.0–10.5. The following buffering systems (50 mM) were utilized: Tris-HCl for the pH 7.0–9.0 range and glycine-NaOH for the pH 9.0–10.5 range. The pH value at which the highest activity was observed and taken as 100%, while other activities were given relatively. The activities of the immobilized WT and Mut preparations were measured at temperatures ranging from 40 to 80 °C to determine their optimum temperatures. The thermal stability of the immobilized WT and Mut preparations was evaluated by incubating them at the determined optimum pH and temperature, followed by measurement of their residual activities. The initial activity of the immobilized WT and Mut preparations was taken as 100%, and their remaining activities were given relatively. The first-order inactivation constant (*k*_*d*_) was determined using the following formula. In biochemical characterization studies, each experiment was repeated at least three times.

First-order inactivation constant (*k*_d_):

3 $$InA_{t}=InA_{0}-k_{d}xt$$

*A*_0_ represents the initial activity, and *At* represents the remaining activity after time *t*.

The half-life (*t*_1/2_) values for the free and immobilized WT and Mut preparations were determined based on their respective inactivation rate constants. These values were calculated using the following equation:

4 $$t_{1/2}=0.693/k_{d}$$

The stabilization factor (SF) was calculated by dividing the half-life of the immobilized WT or Mut preparation at a specific temperature by the half-life of free *Tt*AlaDH at the same temperature.

#### Determination of Kinetic Constants

To determine the kinetic parameters of free and immobilized WT and Mut preparations, activity was measured at the optimum pH and temperature for each sample using substrate concentrations ranging from 0.1 to 20 mM, as described above. The maximum velocity (*V*_*max*_) and Michaelis–Menten constant (*K*_*M*_) of each *Tt*AlaDH preparation were determined by using the GraphPad Prism 9 program. The turnover number (*k*_*cat*_) was calculated using the following formula, where *V*_*max*_ represents the maximum velocity and [*E_T_*] represents the total concentration of active enzyme:

5 $$k_{cat}=V_{max}/\left[E_{T}\right]$$

The catalytic efficiency ratio (CER) was calculated by dividing the *k*_*cat*_/*K*_*M*_ value of the immobilized WT or Mut preparation by the corresponding free WT or Mut preparation.

#### Co-Immobilization of *Tt*AlaDHs and *Ct*FDH

Three different combinations were selected for the coimmobilization of WT or Mut (WT/Mut) and *Ct*FDH (WT/Mut-*Ct*FDH) preparations at different concentrations: Combination 1 (0.25 mg WT/Mut-0.75 mg *Ct*FDH), Combination 2 (0.5 mg WT/Mut-0.5 mg *Ct*FDH), and Combination 3 (0.75 mg WT/Mut-0.25 mg *Ct*FDH). One milliliter of each enzyme combination was mixed with 3 mL of poly­(vinyl alcohol) (PVA) solution. Aliquots (0.1 mL) of these mixtures were pipetted onto a plastic surface and incubated at 5 °C for 12 h to ensure complete gelation. The resulting beads were stored at 5 °C until further use. To determine the optimum amount of immobilized enzyme for synthesis, 10, 25, and 50 mg of coimmobilized WT/Mut-*Ct*FDH preparations were taken. Furthermore, the optimal reaction time was determined by conducting synthesis studies using 25 mg of the coimmobilized WT/Mut-*Ct*FDH preparations for different durations (5–240 min).

#### Asymmetric Synthesis of ncAAs with Coimmobilized WT/Mut-*Ct*FDH

The asymmetric synthesis of l-alanine, L-2-aminobutyrate, l-norvaline, and L-norleucine was performed at the optimal pH and temperature of PVA-WT/*Ct*FDH and PVA-Mut/*Ct*FDH preparations. Briefly, 800 μL of buffer containing 2 M NH_4_Cl, 25 mg of PVA-WT/*Ct*FDH or PVA-Mut/*Ct*FDH preparation, 100 μL of NADH (1 mM), 100 μL of pyruvate, α-ketobutyrate, α-ketovalerate, or α-ketocaproate (100 mM in DMSO), and 100 μL of sodium formate were added to a sealed test tube and incubated at 55 °C for 12 h. After incubation, a 20-μL aliquot was withdrawn and analyzed by high-performance liquid chromatography (HPLC).

#### HPLC Analysis

Unless otherwise stated, all chiral amino acids mentioned in this study refer to the l-enantiomer. Analysis of the synthesized alanine and its derivatives (2-aminobutyrate, norvaline, and norleucine) was performed using a Shodex ORpak CDC-453 HQ chiral column at 205 nm. An acetonitrile/water/acetic acid mixture (20/79/1, v/v/v) was used as the mobile phase at a flow rate of 0.25 mL/min. The oven temperature was maintained at 30 °C during analysis. The enantiomeric excess (%ee) of the synthesized amino acid was calculated using the following formula:

$$\%ee=\frac{\left[S\right]-\left[R\right]}{\left[S\right]+\left[R\right]}\times 100$$

where [*S*] represents the concentration of the S-enantiomer, and [*R*] represents the concentration of the R-enantiomer.

#### Statistical Analysis

All experiments were performed in triplicate, and the data are presented as the mean ± standard deviation (SD). Statistical analyses were conducted using GraphPad Prism 9. Differences between groups were evaluated by one-way analysis of variance (ANOVA), and values of *p* < 0.05 were considered statistically significant.

## Results and Discussion

Noncanonical amino acids such as l-norvaline, L-norleucine, and L-2-aminobutyric acid are valuable building blocks for pharmaceuticals and fine chemicals. Their enzymatic synthesis has been reported using l-AlaDHs, which catalyze the reductive amination of the corresponding α-keto acids with high enantioselectivity.^39^ To enhance the operational and thermal stability of these preparations, immobilization strategies are often employed. Accordingly, the IY% and RA% obtained for the immobilization of WT and Mut preparations are presented in Table 1.

**Table 1:** Summarization Results of IY% and RA% Values for Immobilization of WT and Mut

*Tt*AlaDH	Immobilization type	Added protein amount (mg)	Bound protein amount (mg)	IY (%)	RA (%)
WT	Covalent (EC250L)	9	8.1	90	10.2
Mut	Covalent (EC250L)	9	8.3	92.2	11.5
WT	CLEA	5	4.5	90	5.2
Mut	CLEA	5	4.6	92	5.5
WT	Entrapment (PVA)	1	1	100	95
Mut	Entrapment (PVA)	1	1	100	100

As shown in Table 1, the immobilization yields (IY %) for both WT and Mut preparations on EC250L exceeded 90%. However, the corresponding RA% values were significantly lower, ranging between 10.2% and 11.5%. These relatively low RA% values may be associated with restricted conformational flexibility and increased structural rigidity of TtAlaDH following multipoint covalent attachment to the resin surface, although this interpretation was not directly verified experimentally.^26,28^ For both WT and Mut preparations, approximately 90% of the initially added protein was successfully immobilized by using the CLEA method. However, the RA% values were ∼5%. The low catalytic efficiency may be attributed to excessive structural rigidity resulting from extensive cross-linking, as well as to the formation of dense protein aggregates that restrict substrate accessibility and increase internal mass-transfer resistance.^24^ In contrast, as indicated in Table 1, complete immobilization was achieved for both WT and Mut preparations by using the PVA-based entrapment method. Remarkably, the calculated RA% values ranged from 95% to 100%, indicating the near-total preservation of catalytic activity. These superior results can likely be attributed to the protective microenvironment of the PVA matrix, which allows for greater conformational flexibility and molecular mobility compared with the rigid constraints imposed by covalent attachment or dense cross-linking.^27,40^

Figure 1 shows the effect of pH on the activity of free and immobilized WT (**A**) and Mut (**B**) preparations at 40 °C, respectively. The maximum activity was observed at pH 8.5 for all WTs in Figure 1. Remarkably, EC250L-WT and CLEA-WT exhibited relatively higher activity than the free WT across all tested pH values. These results indicate that covalent immobilization and/or cross-linking of WT and Mut enhance their pH stability.^41,42^ The pH-dependent activity profiles of Mut, EC250L-Mut, CLEA-Mut, and PVA-Mut are presented in Figure 1B. All Mut preparations exhibited maximum activity at pH 9.0. These results demonstrate that the mutations induced a significant shift in the optimum pH toward the alkaline region compared to the WT enzyme. Such a shift suggests that the amino acid substitutions enhanced the structural stability or catalytic efficiency of the enzyme under basic conditions, thereby expanding the industrial applicability of these Mut variants in processes requiring alkaline environments. EC250L-Mut and PVA-Mut displayed nearly identical pH activity profiles, suggesting that the enzyme maintained its structural stability and pH responsiveness throughout immobilization, whereas CLEA-Mut showed higher relative activity across the pH range of 7.0–10.5. This enhanced activity over a broader pH range may be attributed to the greater structural rigidity of CLEA-Mut compared to EC250L-Mut and PVA-Mut.^40,41,43^

**Figure 1:**
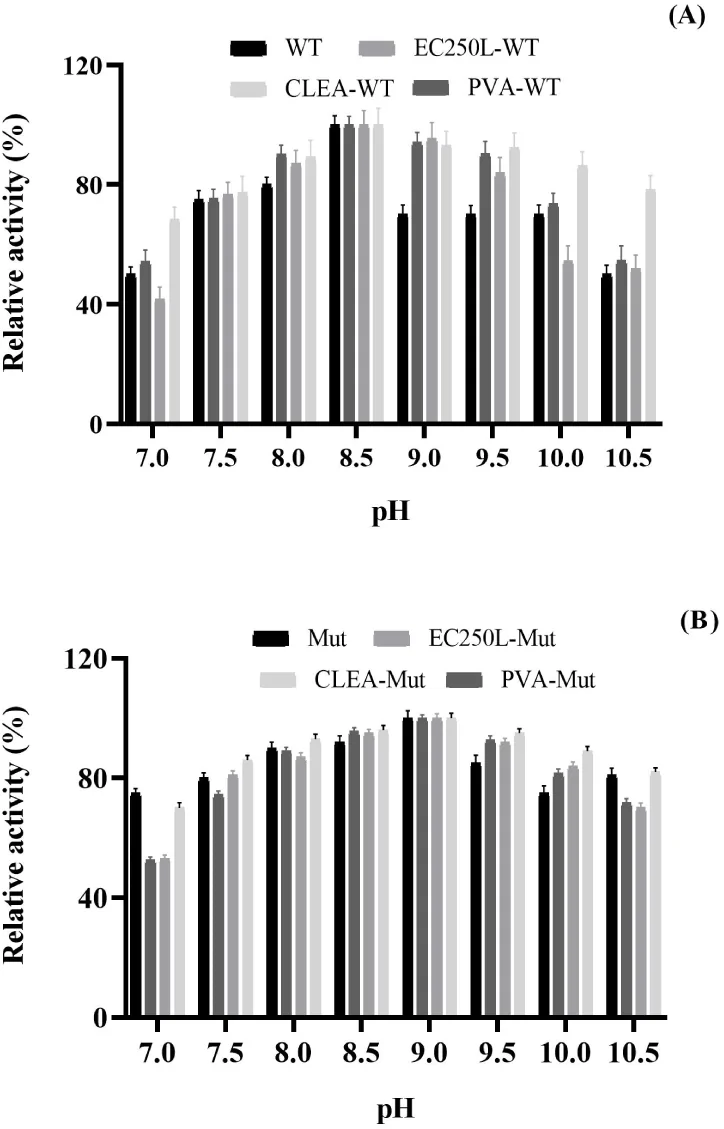
Effect of pH on the reductive amination activity of free and immobilized *Tt*AlaDH preparations. Relative activities of free WT, EC250L-WT, CLEA-WT, and PVA-WT measured at 40 °C over the pH range 7.0–10.5 (A). Relative activities of free Mut, EC250L-Mut, CLEA-Mut, and PVA-Mut under the same conditions (B). Activities were determined using sodium pyruvate as the substrate and expressed relative to the maximum activity observed for each preparation, which was taken as 100%. Data are presented as mean ± SD (n = 3).

The effect of temperature on the activity of free and immobilized WT and Mut preparations at pH 8.5 (A) and at pH 9.0 (B), respectively, was investigated, as presented in Figure 2. Notably, the immobilized WTs exhibited higher relative activity than their free counterparts at 65 °C. Similarly, the optimum temperature of the free Mut enzyme was determined to be 50 °C, whereas the optimum temperature of all immobilized Mut preparations shifted to a higher temperature of 55 °C. These findings suggest that the immobilization matrix provides a stabilizing effect, protecting the enzyme’s tertiary structure against thermal denaturation at elevated temperatures (Figure 2B). Notably, the covalently immobilized variants, EC250L-Mut and CLEA-Mut, showed greater relative activity than PVA-Mut at 65 °C. These results suggest that while the molecular mobility of the Mut enzyme is significantly restricted by covalent attachment, this increased structural rigidity effectively stabilizes the protein scaffold against thermal unfolding at elevated temperatures.^40,41,43^

**Figure 2:**
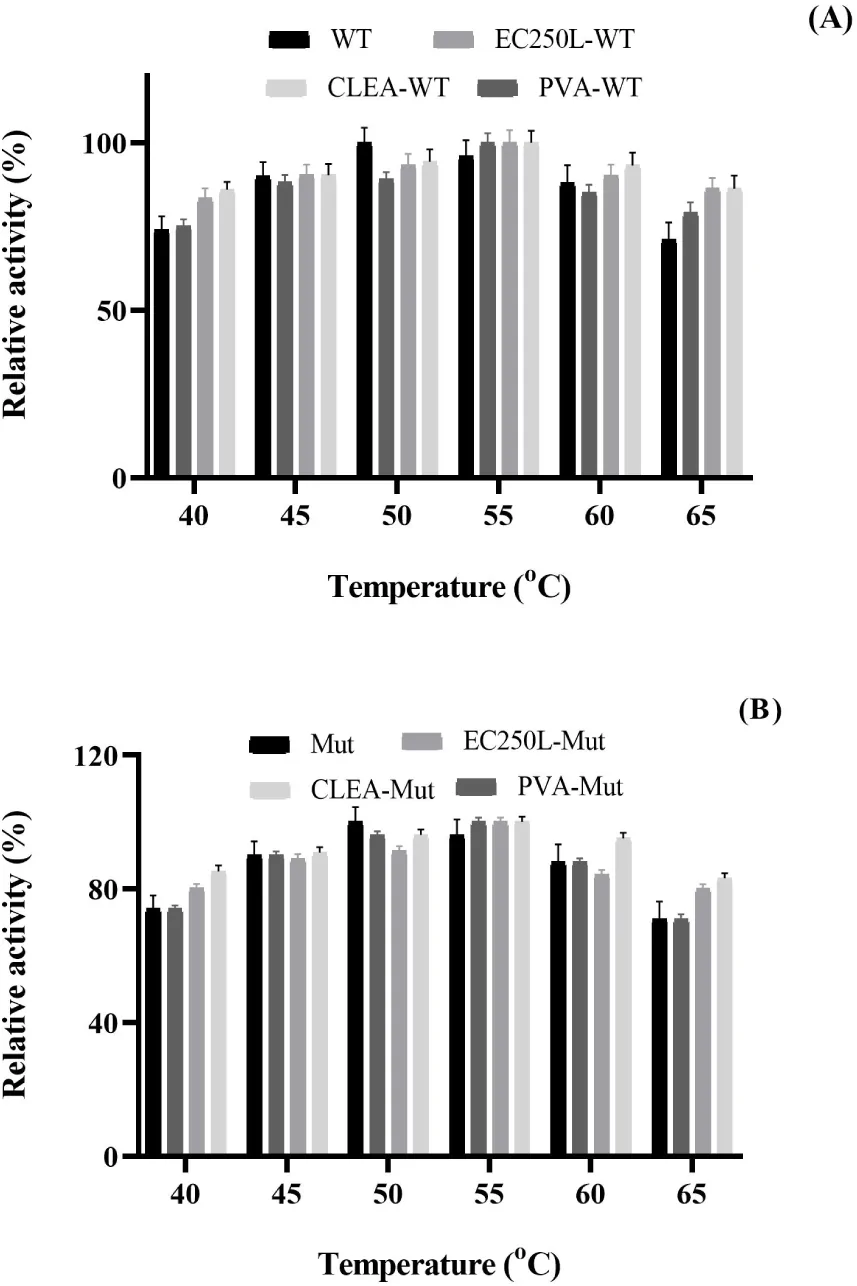
Effect of temperature on the reductive amination activity of free and immobilized *Tt*AlaDH preparations. Relative activities of free WT, EC250L-WT, CLEA-WT, and PVA-WT measured at pH 8.5 over the temperature range 40–80 °C (A). Relative activities of free Mut, EC250L-Mut, CLEA-Mut, and PVA-Mut measured at pH 9.0 under the same conditions (B). Activities were normalized to the maximum activity of each preparation and expressed as relative activity (%). Data are presented as mean ± SD (n = 3).

The thermal stabilities of free WTs preparations and their immobilized preparations EC250L-WT, CLEA-WT, and PVA-WT were investigated at 55 °C (Figure 3A). The free WT showed approximately 50% of its initial activity after 24 h of incubation at 55 °C. The remaining activities were 86%, 87%, and 80% for EC250L-WT, CLEA-WT, and PVA-WT. However, the free Mut enzyme protected almost 60% of its initial activity, whereas the protected activities were approximately 93%, 94%, and 92% for EC250L-Mut, CLEA-Mut, and PVA-Mut, respectively (Figure 3B).

**Figure 3:**
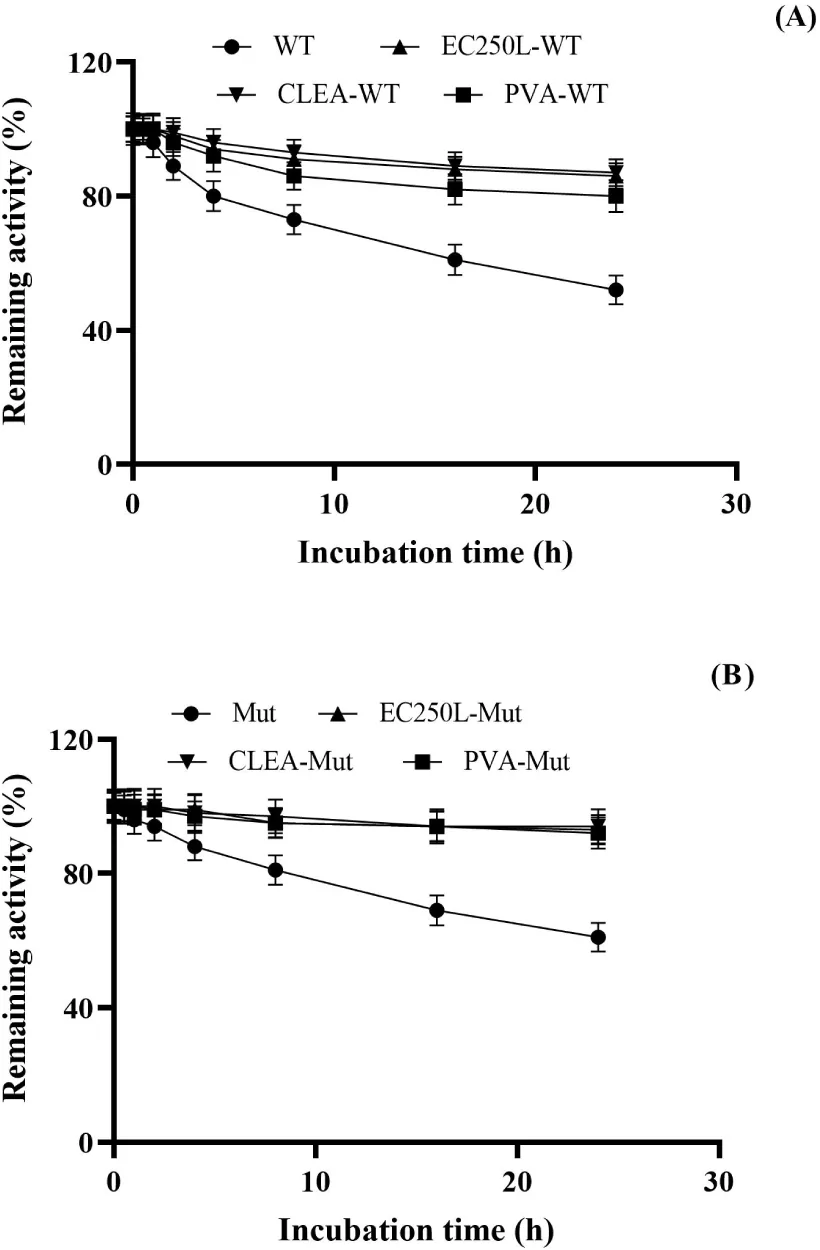
Thermal stability profiles of free and immobilized *Tt*AlaDH preparations. Residual activities of free WT, EC250L-WT, CLEA-WT, and PVA-WT during incubation at pH 8.5 and 55 °C (A). Residual activities of free Mut, EC250L-Mut, CLEA-Mut, and PVA-Mut during incubation at pH 9.0 and 55 °C (B). Residual activities were determined at the indicated time intervals and expressed relative to the initial activity of each preparation, which was taken as 100%. Data are presented as mean ± SD (n = 3).

The *k*_d_ and *t*_1/2_ values of WT and Mut preparations are shown in Table 2. The *k*_*d*_ values were determined to be 3.6 ± 0.2 × 10^–2^, 1.1 ± 0.1 × 10^–2^, 9.6 ± 0.5 × 10^–3^, and 1.6 ± 0.1 × 10^–2^ for free WT, EC250L-WT, CLEA-WT, and PVA-WT, respectively. Their *t*_1/2_ values were calculated as 19.3 ± 1.1, 63.0 ± 3.4, 72.2 ± 4.1, and 43.3 ± 2.5 h. In contrast, the *k*_*d*_ values were found to be 2.7 ± 0.1 × 10^–2^, 5.5 ± 0.3 × 10^–3^, 5.0 ± 0.2 × 10^–3^, and 5.7 ± 0.3 × 10^–3^ h^–1^ for free Mut, EC250L-Mut, CLEA-Mut, and PVA-Mut, respectively. The corresponding *t*_1/2_ values were 25.6 ± 1.3, 126.0 ± 6.8, 138.6 ± 7.2, and 121.6 ± 6.1 h. Based on these results, the SF values showed a 2.2–3.7-fold increase for the immobilized WT preparations and a 4.8–5.4-fold increase for the immobilized Mut preparations. Da Silva et al. observed a 13-fold increase in thermal stability when immobilizing *Bacillus subtilis* l-AlaDH on agarose microbeads activated with glyoxyl groups than the free enzyme.^29^

**Table 2:** *k*_d_ and *t*_1/2_ Values of WT and Mut and Their Immobilized Preparations at 55 °C

*Tt*AlaDH	*k*_d_ (h^–1^)	*t*1/2 (h)	SF
Free (WT)	3.6 ± 0.2 × 10^–2^	19.3 ± 1.1	1
EC250L-WT	1.1 ± 0.1 × 10^–2^	63.0 ± 3.4	3.3
CLEA-WT	9.6 ± 0.5 × 10^–3^	72.2 ± 4.1	3.7
PVA-WT	1.6 ± 0.1 × 10^–2^	43.3 ± 2.5	2.2
Free (Mut)	2.7 ± 0.1 × 10^–2^	25.6 ± 1.3	1.0
EC250L-Mut	5.5 ± 0.3 × 10^–3^	126.0 ± 6.8	4.9
CLEA-Mut	5.0 ± 0.2 × 10^–3^	138.6 ± 7.2	5.4
PVA-Mut	5.7 ± 0.3 × 10^–3^	121.6 ± 6.1	4.8

The *K*_*M*_ values of free and immobilized WT and Mut toward sodium pyruvate are presented in Table 3.

**Table 3:** Kinetic Parameters and Catalytic Efficiency Ratio (CER) of WT and Mut Preparations and Their Immobilized Preparations towards Sodium Pyruvate

l-AlaDH	*K*_*M*_ (mM)	*k*_*cat*_ (s^–1^)	*k*_*cat*_/*K* **_*M*_ (s** ^–1^mM^–1^)	CER
Free (WT)	0.08±0.00	0.97±0.07	12.3±0.08	1.00
EC250L-WT	0.46±0.01	1.09±0.05	2.40±0.05	0.20
CLEA-WT	0.54±0.02	0.36±0.02	0.70±0.03	0.06
PVA-WT	0.18±0.01	1.40±0.03	7.80±0.06	0.63
Free (Mut)	0.44±0.01	7.10±0.1	16.10±0.03	1.00
EC250L-Mut	0.67±0.04	6.80±0.07	10.10±0.06	0.63
CLEA-Mut	0.72±0.03	2.80±0.05	3.90±0.05	0.24
PVA-Mut	0.53±0.03	10.80±0.10	20.40±0.06	1.27

As shown in Table 3, the *K*_*M*_ values of both free and immobilized Mut were higher than those of the corresponding WT preparations. These results indicate a reduced substrate affinity of the Mut for sodium pyruvate. The increased *K*_*M*_ suggests that the mutation negatively affected substrate binding in *Tt*AlaDH. The increase in apparent *K*_*M*_ observed for immobilized enzymes may reflect diffusion limitations or restricted substrate accessibility within the immobilization matrix. However, these mechanisms were not directly investigated in the present study.^44,45^ However, the *k*_*cat*_ values of all Mut preparations were at least 6-fold higher than those of the WT. This increase may result from enhanced stabilization of the transition state, leading to a reduction in activation energy, and/or improved positioning of catalytic residues.^27,40^ When comparing *k*_*cat*_/*K*_*M*_ values, the highest catalytic efficiencies were observed when WT or Mut was immobilized in PVA. In addition, PVA-WT and PVA-Mut exhibited 0.63- and 1.27-fold CER values relative to their respective free preparations. Varan Faki et al.^44^ reported that the immobilization of mannanase in PVA and after cross-linking with genipin increased the CER value by 2.3-fold compared with the free enzyme. Akdoğan and Peksel^46^ showed that the CER value of β-galactosidase increased by 4.5-fold when the enzyme was immobilized in a PVA-based hydrogel.

From an industrial perspective, the coimmobilized enzyme system offers several advantages, including enzyme reuse, reduced cofactor costs through in situ regeneration, and simplified downstream processing. These features highlight the potential applicability of the system in sustainable amino acid production.^25,26,47^ Different combinations of WT or Mut and *Ct*FDH immobilized in PVA, were tested to determine the optimal enzyme system for the production of l-alanine (Figure 4). Using combination 1, l-alanine yields of 52 and 44% were obtained for PVA-WT/*Ct*FDH and PVA-Mut/*Ct*FDH, respectively. When combination 2 was applied, the l-alanine yield increased to 80% for both immobilized enzyme systems. However, the l-alanine yield decreased to 60 and 63% with combination 3 for PVA-WT/*Ct*FDH and PVA-Mut/*Ct*FDH, respectively. In subsequent experiments, immobilized PVA preparations prepared by using combination 2 were selected for further studies.

**Figure 4:**
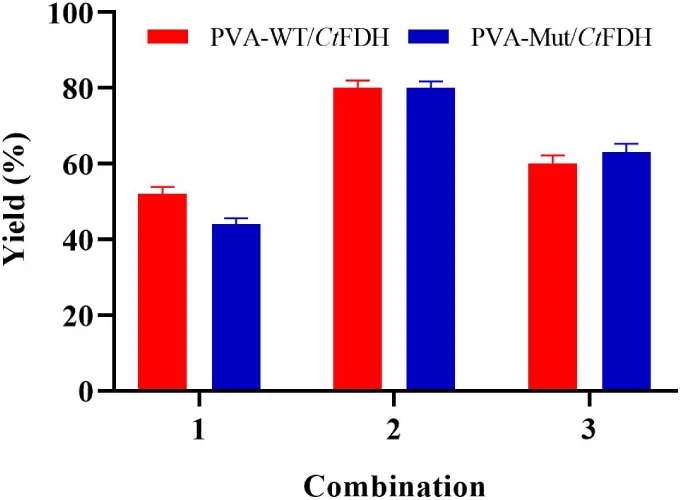
Effect of PVA-WT/*Ct*FDH or PVA-Mut/*Ct*FDH amount for coimmobilization in l-alanine yield. Combination 1 (0.25 mg WT or Mut/0.75 mg *Ct*FDH), Combination 2 (0.5 mg WT or Mut/0.5 mg *Ct*FDH), and Combination 3 (0.75 mg WT or Mut/0.25 mg *Ct*FDH). Data are presented as mean ± SD (n = 3).

To investigate the optimum amount of immobilized enzyme preparation for l-alanine synthesis, 10, 25, and 50 mg of coimmobilized PVA-WT/*Ct*FDH or PVA-Mut/*Ct*FDH preparations were used. When 10 mg of PVA-WT/*Ct*FDH or PVA-Mut/*Ct*FDH was used, the l-alanine yield was in the range of 45% and 41% for PVA-WT/*Ct*FDH and PVA-Mut/*Ct*FDH, respectively (Figure 5). When the biocatalyst loading was increased to 25 mg, the l-alanine yield reached approximately 80% for both the PVA-WT/*Ct*FDH and PVA-Mut/*Ct*FDH systems. However, a decrease in yield was observed upon further increasing the amount of the immobilized enzyme to 50 mg. This decline may be associated with the aggregation of the coimmobilized beads within the reaction medium, potentially leading to mass-transfer limitations and restricted substrate diffusion. However, these effects were not directly examined in this study.^24^ In subsequent studies, 25 mg of coimmobilized WT or Mut/*Ct*FDH preparations were used.

**Figure 5:**
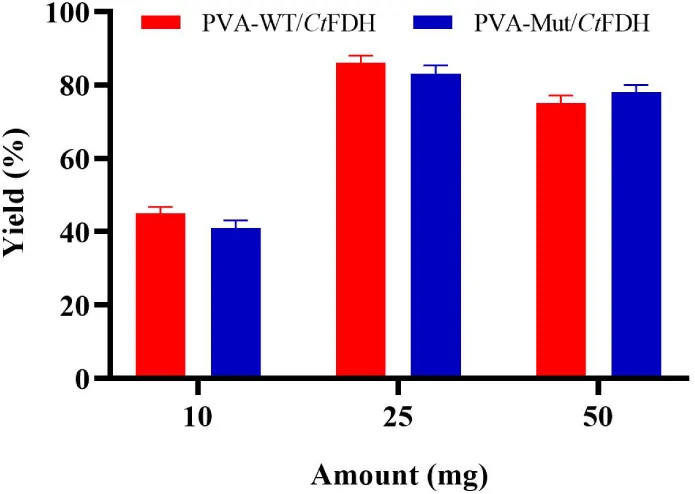
Effect of coimmobilized PVA-WT/*Ct*FDH and PVA-Mut/*Ct*FDH amount on the yield of l-alanine. Data are presented as mean ± SD (n = 3).

To determine the optimum reaction time for l-alanine synthesis, 25 mg of coimmobilized WT or Mut/*Ct*FDH preparations were used, and 1 mL of reaction mixture containing 100 mM sodium pyruvate, 1 mM NADH, and 100 mM ammonium formate at the determined optimum pH and temperature was added. Twenty microliter of the reaction mixture was taken for different reaction times, and the product yield was determined by HPLC (Figure 6). It was determined that the amount of l-alanine formed increased linearly between 5 and 30 min, while the l-alanine yield did not change significantly between 120 and 240 min. After 120 min, the l-alanine yields obtained for PVA-WT/*Ct*FDH and PVA-Mut/*Ct*FDH were 87% and 83%, respectively. Consequently, a reaction time of 120 min was selected for all subsequent synthesis experiments to ensure a maximum productivity.

**Figure 6:**
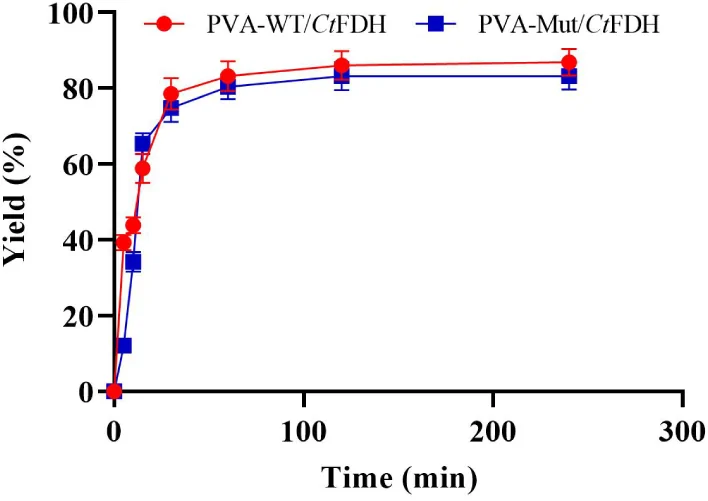
Time-course synthesis of l-alanine catalyzed by PVA-WT/*Ct*FDH and PVA-Mut/*Ct*FDH. Data are presented as mean ± SD (n = 3).

Figure 7 shows the synthesis of L-2-aminobutyrate, l-norvaline, and L-norleucine catalyzed by PVA-WT/*Ct*FDH and PVA-Mut/*Ct*FDH after 120 min of reaction time. As illustrated in Figure 7, PVA-WT/*Ct*FDH exhibits significantly higher selectivity toward L-2-aminobutyrate, whereas PVA-Mut/*Ct*FDH demonstrates greater selectivity for l-norvaline and L-norleucine. The yields of L-2-aminobutyrate, l-norvaline, and L-norleucine obtained with PVA-WT/*Ct*FDH were 63.9, 11.8, and 8.6%, respectively. In comparison, the corresponding yields achieved with PVA-Mut/*Ct*FDH were 42.6, 47.5, and 74.8%, respectively. The distinct selectivity observed for PVA-WT*Ct*FDH and PVA-Mut/*Ct*FDH is likely attributed to differences in the substrate-binding pocket and steric environment of the WT and Mut variants, which influence their substrate preference and catalytic efficiency toward different amino acid products.^27,48^ This behavior is consistent with our previous structural analysis of the Tyr92Ser mutant, where replacement of the bulky tyrosine residue with the smaller serine residue enlarged the substrate-binding pocket and reduced steric hindrance around the active site.^20^ This structural modification likely facilitates accommodation of bulkier α-keto acid substrates such as α-ketovalerate and α-ketocaproate, resulting in enhanced synthesis of l-norvaline and L-norleucine. Meanwhile, the ee values of L-2-aminobutyrate, l-norvaline, and L-norleucine were all 99% (Figure S1). These findings suggest that immobilized Mut could be used as a key process in the reductive amination pathway for the synthesis of ncAAs derivatives.

**Figure 7:**
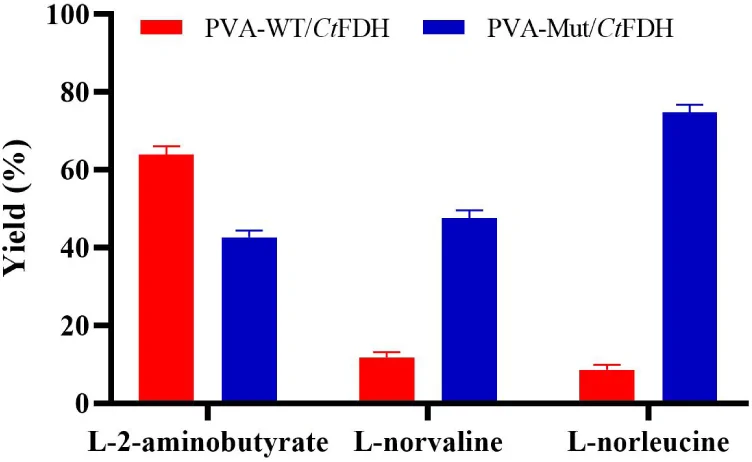
Synthesis of L-2-aminobutyrate, l-norvaline, and L-norleucine catalyzed by PVA-WT/*Ct*FDH and PVA-Mut/*Ct*FDH after 120 min of reaction time. Data are presented as mean ± SD (n = 3).

The synthesis and production of biocatalysts entail large costs. Therefore, the implementation of immobilization strategies is required to ensure the economic feasibility of processes that require extended operational stability and repeated catalytic use. The reuse stability of PVA-WT/*Ct*FDH and PVA-Mut/*Ct*FDH was investigated for l-alanine synthesis in a batch reactor. The changes in activity of PVA-WT/*Ct*FDH and PVA-Mut/*Ct*FDH depending on repeat use are shown in Figure 8. The operational stability of the coimmobilized systems was evaluated over multiple reaction cycles. After five consecutive reuses, PVA-WT/*Ct*FDH and PVA-Mut/*Ct*FDH retained 80% and 84% of their initial activities, respectively. Evaluation beyond five reuse cycles was not performed because the PVA hydrogel beads became increasingly swollen during repeated use, making their recovery from the reaction medium more difficult and resulting in material loss during handling. Consequently, the long-term operational stability beyond five cycles could not be reliably assessed. In comparison, Nakane et al.^47^ reported a significantly lower residual activity of 60% after 6 cycles for l-AlaDH from *Bacillus stearothermophilus* immobilized on cellulose acetate-zirconium alkoxide hybrid gel fibers. The minimal activity loss suggests that enzyme leaching from the PVA matrix was negligible, indicating that the entrapment system provided effective structural stabilization of the enzymes during repeated catalytic cycles.

**Figure 8:**
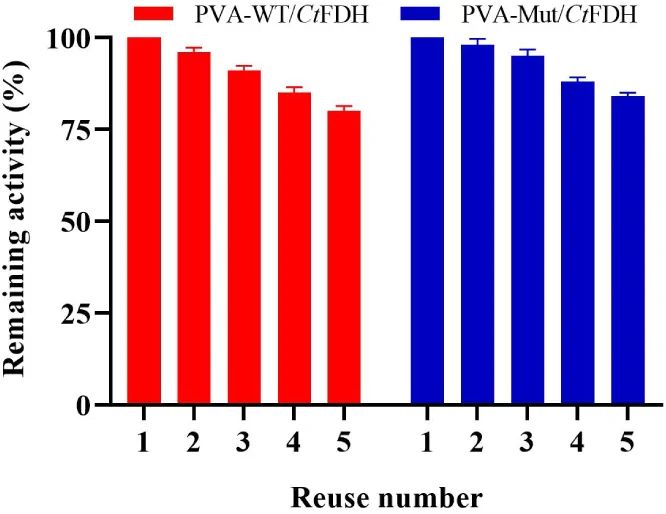
Reuse stability of PVA-WT/*Ct*FDH and PVA-Mut/*Ct*FDH for l-alanine synthesis. Data are presented as mean ± SD (*n* = 3).

Compared with previously reported immobilized l-alanine dehydrogenase systems, the present biocatalyst exhibits several notable advantages. Immobilization of *Bacillus subtilis* l-AlaDH on glyoxyl-activated agarose has primarily focused on improving enzyme stability, while cellulose acetate–zirconium hybrid fibers were employed for l-alanine production with cofactor regeneration.^29,47^ More recently, immobilization of *Tt*AlaDH on magnetic nanoparticles was reported to improve enzyme stability and reusability.^32^ In contrast, the present study systematically compares three immobilization strategies for both wild-type and engineered *Tt*AlaDH variants and demonstrates that PVA entrapment provides the best balance between immobilization yield, activity retention, and thermal stability. Furthermore, coimmobilization with *Ct*FDH enabled efficient in situ NADH regeneration and the asymmetric synthesis of several noncanonical amino acids with excellent enantiomeric excess (99% ee). To the best of our knowledge, this is the first study integrating protein engineering, comparative immobilization, and coimmobilized cofactor regeneration into a reusable platform for the synthesis of l-alanine-derived noncanonical amino acids.

## Conclusion

This study demonstrates an effective and sustainable strategy for producing enantiopure noncanonical L-amino acids through a one-pot biocatalytic cascade. WT and Mut were immobilized using covalent attachment, CLEA formation, and PVA entrapment, and the results indicated that the immobilization method fundamentally dictates catalytic performance. Although covalent immobilization and CLEA preparation provided high immobilization yields, they caused severe losses in recovered activity, likely due to conformational restriction and diffusion limitations. In contrast, PVA entrapment preserved near-complete catalytic activity while significantly enhancing thermal stability. This indicates that the PVA hydrogel provides a more biomimetic microenvironment that maintains enzyme flexibility, making it the most suitable approach for designing the integrated biocatalytic cascade. By coimmobilizing WT and Mut with *Ct*FDH in PVA gel, an efficient NADH regeneration system was integrated directly into the biocatalyst, enabling practical reductive amination of α-keto acids under mild conditions. The spatial proximity of WT (or Mut) and *Ct*FDH within the PVA matrix may contribute to improved cofactor recycling by reducing the diffusion distance of NADH between the enzymes. Although this interpretation is consistent with the observed catalytic performance and previous reports, it was not directly demonstrated in the present study.

The optimized PVA-WT/*Ct*FDH and PVA-Mut/*Ct*FDH systems produced l-alanine at high yield (up to 87%) and enabled the synthesis of several artificial amino acids with excellent enantiomeric excess (99% ee). Notably, the Mut variant showed a distinct substrate preference, allowing improved yields of l-norvaline and L-norleucine compared with the WT enzyme. In addition, the coimmobilized preparations maintained most of their activity after repeated batch reuse, highlighting their operational stability. Overall, the combination of enzyme engineering, PVA-based immobilization, and cofactor regeneration through coimmobilized FDH provides a robust platform for the scalable synthesis of valuable chiral amino acids.

By overcoming the limitations of traditional immobilization and expanding the enzyme’s substrate scope, this approach achieves a high degree of atom economy and operational efficiency. Notably, this work represents the first comprehensive investigation into the high-efficiency biocatalytic synthesis of l-alanine-derived ncAAs through the synergy of protein engineering and multienzyme coimmobilization. Future studies will focus on improving the mechanical stability of the PVA matrix to minimize swelling during repeated use, thereby enabling extended reuse studies and a more comprehensive evaluation of long-term operational stability.

## Supplementary Material



## Data Availability

Data will be made available upon reasonable request.

## Notes

**Figure S1.** Chiral HPLC chromatograms of l-alanine, L-2-aminobutyrate, l-norvaline, and L-norleucine; supplementary experimental details (PDF)
