# Supplementary material for: Immobilized Wild-Type and Mutant L‑Alanine Dehydrogenase from Thermus thermophilus Coupled with Formate Dehydrogenase for Asymmetric Synthesis of Noncanonical Amino Acids
Source: ACS Omega. 2026 Aug 19;11(36):54068–76. doi: 10.1021/acsomega.6c04318 (PMC13584793; doi:10.1021/acsomega.6c04318)
Supplement: Supplementary file 1 [file ao6c04318_si_001.pdf]

Supporting information

Immobilized wild-type and mutant L-alanine dehydrogenase from *Thermus thermophilus* coupled with formate dehydrogenase for asymmetric synthesis of non-canonical amino acids

Ğarip Demir[a], Deniz Yildirim[b*] and Barış Binay[c,d*]

[a] Department of Molecular Biology and Genetics, Gebze Technical University, 41400, Gebze, Kocaeli, Türkiye

[b] Department of Chemical Engineering, Faculty of Engineering, University of Çukurova, 01130, Adana, Türkiye

[c] Department of Bioengineering, Faculty of Engineering, Gebze Technical University, 41400, Gebze, Kocaeli, Türkiye

[d] BAUZYME Biotechnology Co., Gebze Technical University Technopark, 41400, Gebze, Kocaeli, Türkiye

***Co-corresponding author: Prof. Dr. Deniz Yıldırım**

Department of Chemical Engineering, Faculty of Engineering, University of Çukurova, 01330, Adana, Türkiye, Tel: +90 (322) 3386081/25

E-mail: dyildirim@cu.edu.tr

***Co-corresponding author: Prof. Dr. Barış Binay**

Department of Bioengineering, Faculty of Engineering, Gebze Technical University, 41400, Gebze, Kocaeli, Türkiye, Tel: +90 (262) 605 22 80

E-mail: binay@gtu.edu.tr

HPLC analysis

Unless otherwise stated, all chiral amino acids mentioned in this study refer to the L-enantiomer. Analysis of the synthesized alanine and derivatives (2-aminobutyrate, norvaline, and norleucine) was performed using a Shodex ORpak CDC-453 HQ chiral column at 205 nm. An acetonitrile/water/acetic acid mixture (20/79/1, v/v/v) was used as the mobile phase at a flow rate of 0.25 mL/min. The oven temperature was maintained at 30°C during analysis. The retention times of L-alanine, L-2-aminobutyrate, L-norvaline, and L-norleucine are 3.9, 4.25, 5.4, and 3.8 min, respectively.

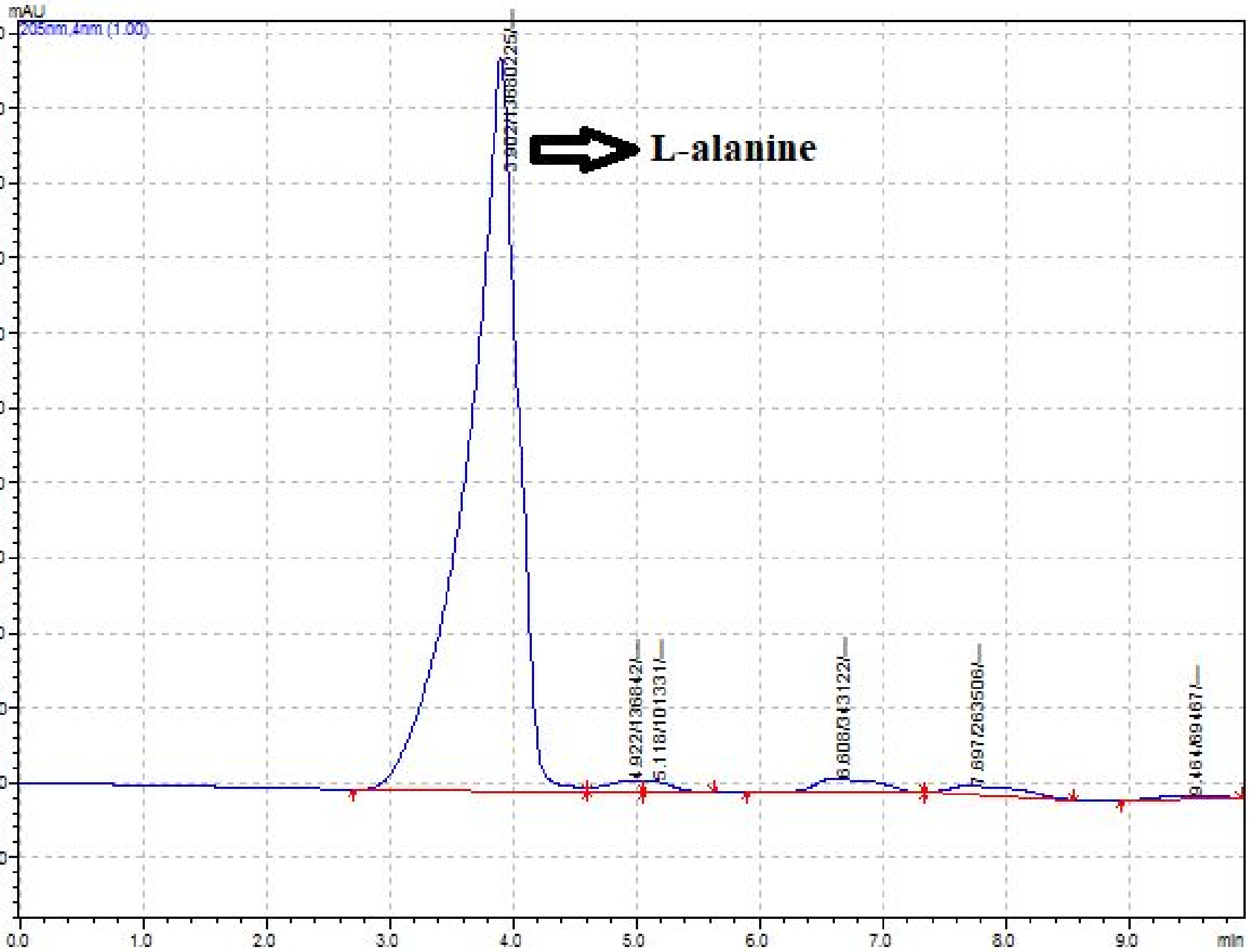

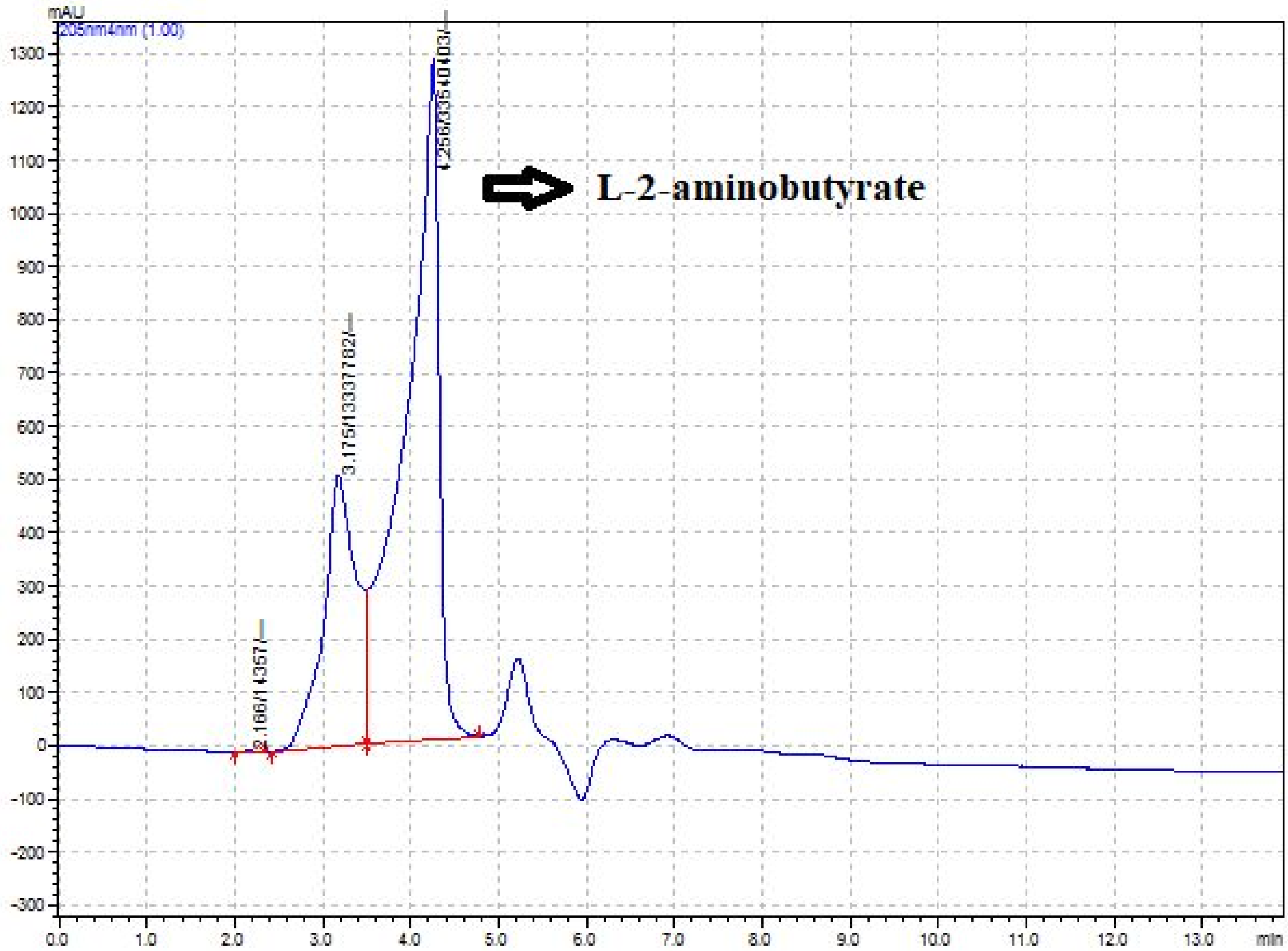

mAU
205nm4nm (1.00)
L-2-aminobutyrate
min

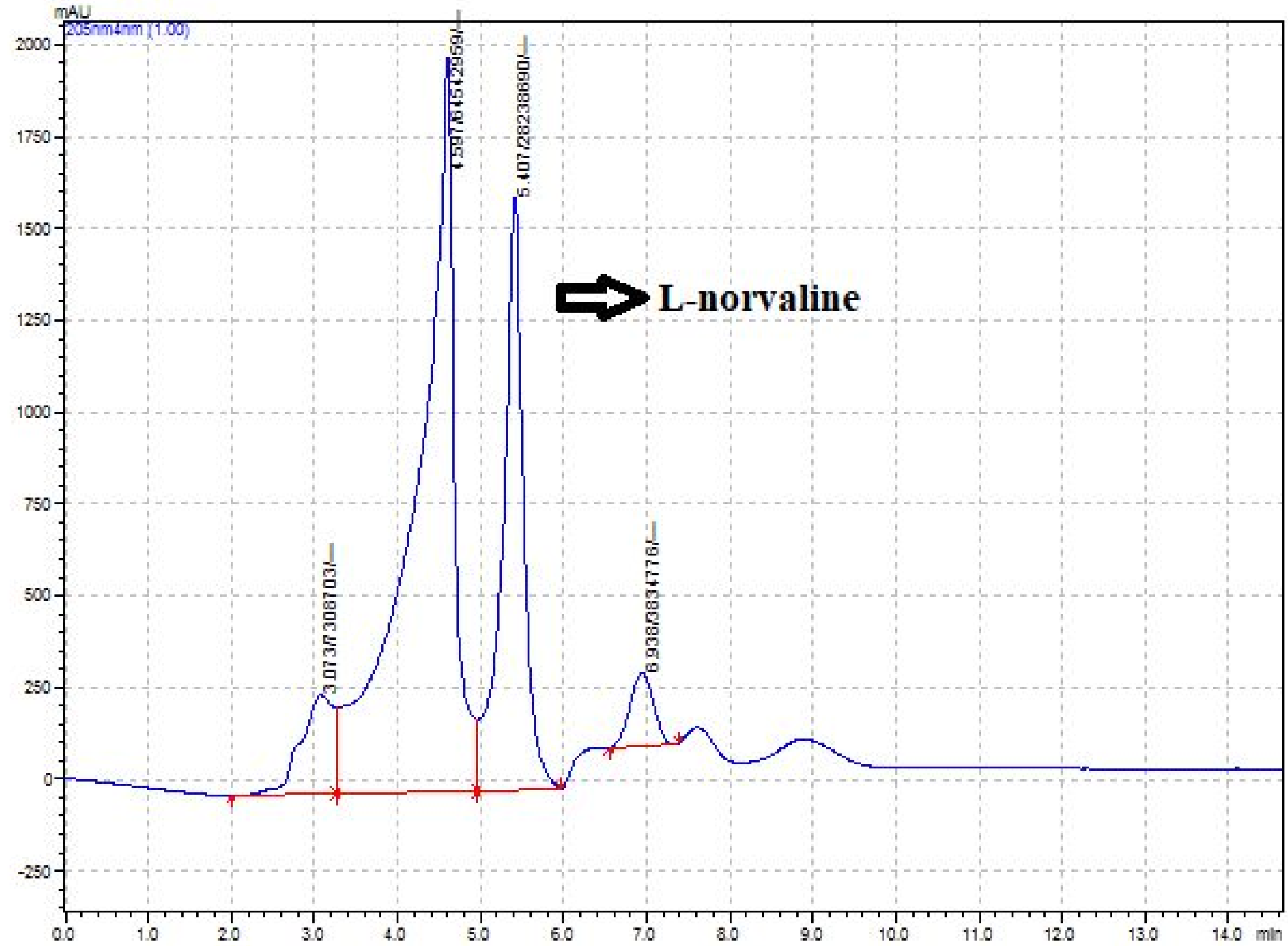

mAU
205nm4nm (1.00)
L-norvaline
min

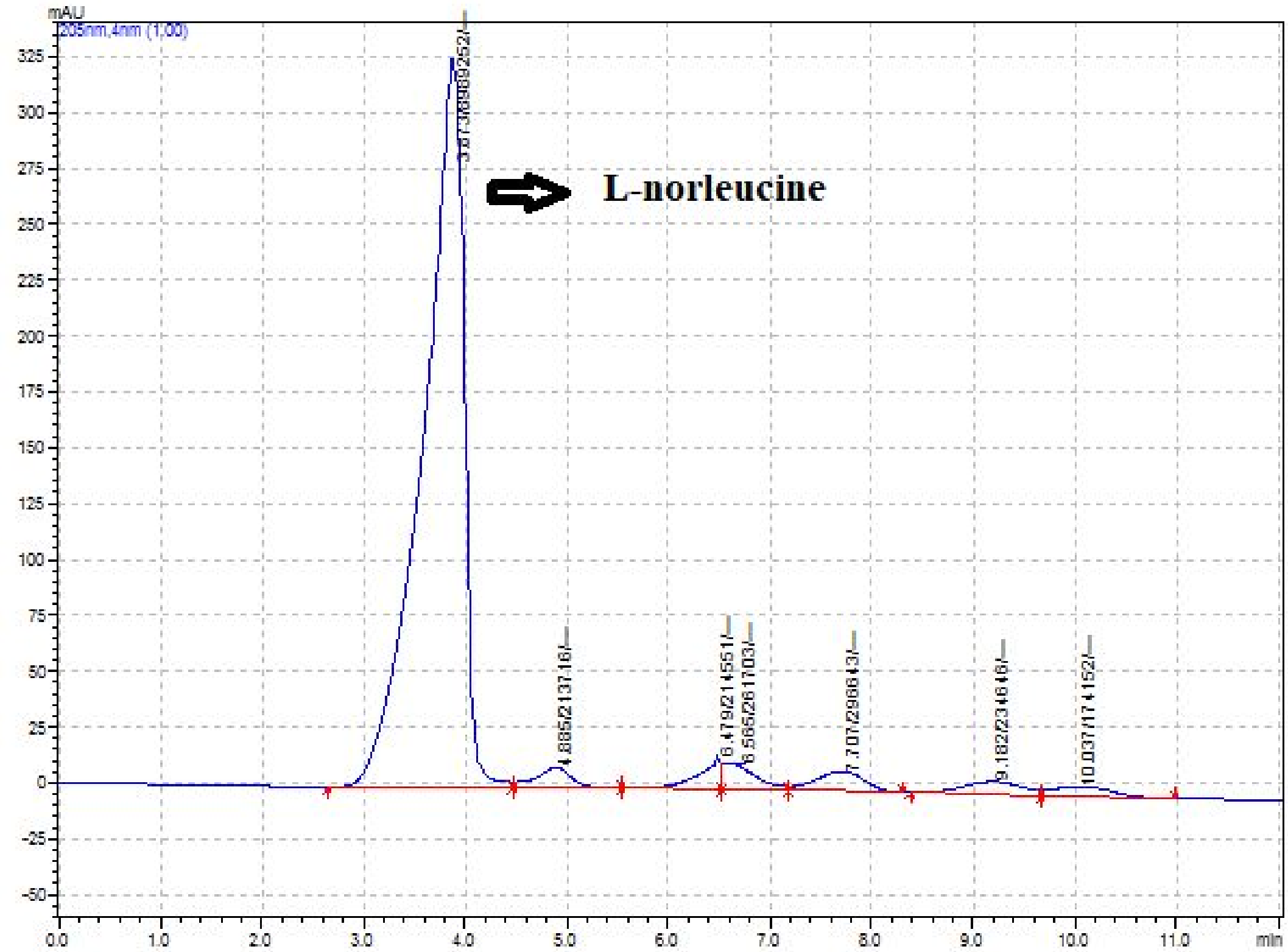

Fig. S1. Chiral HPLC chromatograms of L-alanine, L-2-aminobutyrate, L-norvaline, and L-norleucine.
